# Alexithymia in multiple sclerosis: past, present and future

**DOI:** 10.3389/fnhum.2025.1552494

**Published:** 2025-02-07

**Authors:** Samar S. Ayache, Moussa A. Chalah

**Affiliations:** ^1^Department of Neurology, Gilbert and Rose-Marie Chagoury School of Medicine, Lebanese American University, Byblos, Lebanon; ^2^Institut de la Colonne Vertébrale et des NeuroSciences (ICVNS), Centre Médico-Chirurgical Bizet, Paris, France; ^3^EA4391 Excitabilité Nerveuse & Thérapeutique, Université Paris-Est Créteil, Créteil, France; ^4^Department of Clinical Neurophysiology, DMU FIxIT, Henri Mondor University Hospital, Assistance Publique-Hôpitaux de Paris (APHP), Créteil, France; ^5^Institut de Neuromodulation, Service Hospitalo-Universitaire, Pôle Hospitalo-Universitaire Psychiatrie Paris 15, Hôpital Sainte-Anne, GHU Paris Psychiatrie et Neurosciences, Paris, France

**Keywords:** multiple sclerosis, alexithymia, neurobiology, fatigue, depression, anxiety, interoception, psychotherapy

## Abstract

Alexithymia denotes the “absence” of “words” for “emotion” and has its roots in the Greek words “a,” “lexis,” and “thymos.” It is sometimes referred to as “emotional blindness,” “blunted feeling,” or “disrupted emotional awareness.” The term “alexithymia” first appeared in the 1970s in the works of Sifneos, Nemiah, and colleagues. It entails difficulties in identifying and expressing emotions and an externally oriented thinking style. It is not a psychiatric disorder but rather a multidimensional personality trait or construct, appearing to be normally distributed in the general population, with high levels of alexithymia in approximately 10% of individuals. Evidence suggests that alexithymia serves as a prognostic risk factor for health problems, a transdiagnostic risk factor for emotion-based psychopathologies, and a predictor of poor psychiatric treatment outcomes. It is frequently observed in neurological diseases. Nevertheless, its mechanisms, assessment, and management remain overlooked. In multiple sclerosis (MS), an autoimmune disease of the central nervous system, alexithymia seems to occur in up to 53% of patients. However, it remains understudied despite recent growing interest. In this mini review, we briefly reassess the prevalence, as well as the clinical, sociodemographic and neuropsychological correlates of alexithymia in MS (e.g., anxiety, depression, fatigue, socio-emotional outcomes). This is followed by an analysis of neurobiological underpinnings of alexithymia derived from neurophysiological and neuroimaging studies in this clinical population. Finally, we provide perspectives to guide future research exploring and managing alexithymia in MS.

## 1 Introduction

Alexithymia, a multidimensional personality construct, denotes the “absence” of “words” for “emotion” and finds its roots in the Greek language: “a,” “lexis” and “thymos.” Also sometimes associated with or referred to as “emotional blindness” (Becerra et al., 2002), “blunted feeling” (Goerlich-Dobre et al., 2014b), “affective agnosia” (Lane et al., 2015), “disrupted emotional awareness” (Hogeveen and Grafman, 2021), “impairment in the mental representation of emotions” (Taylor et al., 2016), the term “alexithymia” first appeared in the works of the analysts Sifneos and Nemiah involving patients with psychosomatic disorders (Nemiah and Sifneos, 1970; Sifneos, 1973). Alexithymia is not a psychiatric disorder but rather a personality trait or construct that entails four components: difficulty identifying feelings (DIF) and distinguishing them from bodily sensations of emotional arousal, difficulty describing feelings (DDF), externally oriented thinking, and paucity of imaginal processes/fantasy life (Taylor et al., 2024).

It seems to be normally distributed in the general population with high levels occurring in 10% of individuals (Parker et al., 2008). However, some evidence suggests it to be a prognostic risk factor for somatic and psychiatric health problems (Kojima, 2012; Hemming et al., 2019), a transdiagnostic risk factor for emotion-based psychopathologies (Preece et al., 2024), as well as poor psychiatric treatment outcomes (Pinna et al., 2020). It can be evaluated using dedicated scales, the two most employed ones being the Toronto Alexithymia Scale (Taylor et al., 1985) and Bermond-Vorst Alexithymia Questionnaire (Vorst and Bermond, 2001).

Alexithymia is frequently observed in neurological diseases (Ricciardi et al., 2015). It seems to be particularly frequent in multiple sclerosis (MS), an autoimmune disease of the central nervous system with a spectrum of cognitive, affective and psychiatric manifestations (Grigorescu et al., 2023). Yet, it remains understudied with only recent growing interest in this topic. Its further understanding would help improve its management and optimize the patients’ quality of life. In this mini review, the prevalence, as well as clinical and neuropsychological correlates of alexithymia in MS, will be briefly reappraised. This will be followed by an analysis of the neurobiological underpinnings of alexithymia in this clinical population. Finally, this article provides some suggestions that would help guide future research that aims to explore and manage alexithymia in MS.

## 2 Prevalence and clinical and neuropsychological correlates of alexithymia in MS

A previous review showed that alexithymia seems to occur in 10–53% of patients with MS (PwMS) (Chalah and Ayache, 2017). More recent works are line with these data reporting alexithymia in 24.2–51.7% of patients (Eboni et al., 2018; Chalah et al., 2020a, 2020b; Stojanov and Stojanov, 2020; Capet et al., 2021; Karpuz Seren et al., 2022; Taskin Yilmaz et al., 2023; Joly et al., 2024). One work reported a higher prevalence but included cases of borderline alexithymia (van Assche et al., 2021). Also, alexithymia prevalence reaches 29–42% in clinically isolated syndrome (CIS) (Capet et al., 2021; Jougleux et al., 2021) and 34% in radiologically isolated syndrome (RIS) (Joly et al., 2024).

Sociodemographic and clinical variables do not appear to be associated with alexithymia in PwMS (for review see Chalah and Ayache, 2017; Chalah et al., 2020a, 2020b; van Assche et al., 2021; Karpuz Seren et al., 2022). However, a few works suggest a relationship between alexithymia and young or old age, low educational level, single or with few children, unemployment, disability, number of relapses, disease duration, and primary progressive type (Chahraoui et al., 2008; Dulau et al., 2017; Eboni et al., 2018; Chalah et al., 2020a, 2020b; Stojanov and Stojanov, 2020).

The relationship between alexithymia, anxiety, depression and fatigue has been explored in several works. The concept of ‘symptoms cluster’ has been suggested to characterize the co-occurrence of several MS symptoms that might interact with each other and share common pathophysiological pathways (Ayache and Chalah, 2024). With some exceptions (e.g., Dulau et al., 2017; Karpuz Seren et al., 2022), studies suggest alexithymia to be associated with depression, anxiety and fatigue in PwMS (Chahraoui et al., 2014; Chalah and Ayache, 2017; Eboni et al., 2018; Chalah et al., 2019, 2020b; Christopoulos et al., 2020; Pust et al., 2020; Stojanov and Stojanov, 2020; Capet et al., 2021; van Assche et al., 2021; Carvalho et al., 2023), even in CIS or RIS (Jougleux et al., 2021; Joly et al., 2024).

Some data suggest emotional regulation difficulties and employment of less efficient coping strategies among PwMS. Very early work has documented an association between alexithymia and blunt affect (Montreuil and Lyon-Caen, 1993). In addition, an association was found between alexithymia and difficulties in emotion regulation (Mosson et al., 2014; Gay et al., 2017). With regards to coping strategies, few works done found an association between alexithymia and frequent employment of avoidance or instinctive coping (Briones-Buixassa et al., 2019; Pust et al., 2021), but less frequent seeking of social support (Briones-Buixassa et al., 2019) and well-being (Gay et al., 2017), and less adoption of problem-focused coping (Taskin Yilmaz et al., 2023).

Concerning general cognition, many studies did not assess it, nor study its relationship with alexithymia (Chalah and Ayache, 2017). The very few available data suggest no relationship between alexithymia and general cognitive deficits in MS, CIS or RIS (Dulau et al., 2017; Chalah et al., 2020a, 2020b; Jougleux et al., 2021; Karpuz Seren et al., 2022; Joly et al., 2024). Conversely, alexithymia was inversely correlated with information processing speed in one work (Capet et al., 2021) and tended to correlate with it in another work (Chalah et al., 2020b).

Few available works hint towards the existence of a relationship between alexithymia and some social cognitive domains. Facial emotion recognition is not associated with alexithymia in MS (Prochnow et al., 2011; Cecchetto et al., 2014; Pfaff et al., 2021). As for theory of mind, the available works yielded inconsistent findings which might be related to the inter-study clinical and sociodemographic differences (Chalah et al., 2017a, 2017b; Raimo et al., 2017; Karpuz Seren et al., 2022). Chalah *et al.* recruited patients with progressive MS subtypes, whereas Raimi et al. and Karpuz Seren et al., included younger and less disabled patients with exclusively or predominantly relapsing remitting (RR) MS and shorter disease duration. Finally, few works concomitantly assessed alexithymia and empathy reporting an inverse correlation between these variables in PwMS (Gleichgerrcht et al., 2015; Chalah et al., 2017a,b, 2020a).

Besides social cognition, moral judgment has been the subject of even fewer works (Gleichgerrcht et al., 2015; Patil et al., 2017; Ayache and Chalah, 2018). In PwMS, one work found an association between alexithymia and altered moral judgment (Gleichgerrcht et al., 2015). Ongoing research further explores this relationship, and the results are awaited (Zikos et al., 2024).

## 3 Neurobiological correlates of alexithymia in MS

First, the hypothesis of a deficit in interhemispheric transfer in the context of alexithymia was raised more than 30 years ago when studying patients with split-brain (Chalah and Ayache, 2017). In MS, aberrant interhemispheric transfer and pathologies involving the corpus callosum (CC) have been previously reported (Chalah and Ayache, 2017). Montreuil and Lyon-Caen documented a dissociation between patients’ subjective emotional experience and clinically perceived emotional expression (Montreuil and Lyon-Caen, 1993). Their cohort’s performance on a neuropsychological dual task that assesses interhemispheric transfer of information was correlated with alexithymia, with higher ratings associated with worse performance.

The neurophysiological correlates of alexithymia were assessed in only one work involving 27 PwMS (progressive types) using transcranial magnetic stimulation (TMS) (Chalah et al., 2020b). Here, patients expressing high alexithymia scores had shorter cortical silent period (CSP) compared to those with low scores, and an inverse correlation was found between alexithymia scores and CSP, reflecting a defective cortical GABAergic inhibition. These results converge with other works involving patients with borderline personality disorder (Lang et al., 2011).

Three studies have included structural MRI when exploring alexithymia in MS. In the first study involving a small cohort of PwMS and moderate disability, an inverse correlation was found between the posterior callosal volume and alexithymia scores (Pelletier et al., 1996). In the second study including 45 PwMS (progressive types), patients with high alexithymia ratings had lower volumes of CC, pallidum, thalamus (left) and deep white matter (Chalah et al., 2020a). In addition, an inverse correlation was found between alexithymia scores and each of thalamic, pallidal, callosal and deep white matter volumes. In the third work comprising 95 PwMS (different disease types), an inverse correlation was found between alexithymia scores and thalami, brainstem, CC, cerebellar and cerebral white matter, but not with T2 lesion volume (Capet et al., 2021). However, some findings were only significant within specific disease phenotypes. For instance, the relationship with the white matter atrophy was significant in patients with early disease process (CIS and RR MS). In addition, the relationship with thalamic and brainstem atrophy was only significant in the CIS subgroup.

Finally, two studies employed functional MRI (fMRI). The first one included 25 PwMS (RR) and 27 healthy participants, and conducted fMRI during the visualization of emotional scenes with different valences and arousals after which the participants rated the emotion valence and arousal sensation (Pfaff et al., 2019). Compared to healthy controls, patients had higher alexithymia ratings, and exhibited a more scattered emotional experience, a higher variability of responses in the left orbital inferior frontal gyrus (IFG) for positive stimuli, and a trend to a higher variability in other brain areas for negative stimuli (left amygdala, right fusiform gyrus, right caudate nucleus, right pallidum) (Pfaff et al., 2019). Alexithymia, structural or functional cerebral dysconnectivity, might account for the observed brain variability among patients during emotional tasks. However, the correlation between alexithymia scores and the activity of the left orbital IFG was not significant and subgroup analyses according to alexithymia scores or connectivity pattern were not considered (Pfaff et al., 2019). In the second work, 19 PwMS (RR) and 20 healthy controls underwent fMRI during the visualization of scenes conveying different emotions and rated the intensity of their emotional state right after viewing versus after cognitive reappraisal and emotion regulation (van Assche et al., 2021). Patients expressed hyperactivation in ventral prefrontal areas (orbital IFG, subgenual ACC, and left caudate nucleus) compared to controls regardless of the conditions. In addition, they exhibited a hyperconnectivity between the orbital IFG and the amygdala, during cognitive reappraisal of negative scenes, which was directly correlated with alexithymia (DIF subscale). The latter findings suggest a deficient downregulation of amygdalar (limbic) activity during the reappraisal of negative emotions in PwMS exhibiting high alexithymia scores (van Assche et al., 2021). The recruitment of frontostriatal circuits in PwMS might reflect a compensatory mechanism aiming to preserve amygdalar homeostasis.

The incriminated brain regions are known to be involved in the neural circuitry of alexithymia (Messina et al., 2014; Goerlich-Dobre et al., 2014a, 2014b, 2015; Xu et al., 2018). The findings altogether could be interpreted in the light of the cognitive development of emotions (LeDoux and Bemporad, 1997). Here, the authors suggest two cerebral pathways for the emotional experience: (1) A direct and unconscious low road that oversees triggering fast emotion-related autonomic and endocrine responses and passes through the brainstem, thalamus and hypothalamus, and (2) an indirect high road involving complex cognitive mechanisms of the representation and memorization and including the thalamus, prefrontal neocortex as well as paralimbic structures. In this model, the white matter tracts would ensure communication between the mentioned hubs. The integration of emotional stimuli arising from both pathways into working memory would result in a conscious emotional experience (Kano and Fukudo, 2013).

The findings could be also discussed by considering the alexithymia model presented by Bermond and colleagues (Bermond et al., 2006). The model involves the affective and the cognitive dimensions of alexithymia, and suggests an interconnection and reciprocal influence among their underlying cerebral components. While the former seems to incriminate the orbito/medial-prefrontal cortices, the latter seems to implicate right temporal cortex areas which also contribute to the former via their links with the orbito-prefrontal cortices. Interhemispheric white matter (i.e., CC) ensures the transfer of information from right-hemispheric regions (related to global nonverbal overview of emotions) to the left-hemispheric regions (in charge of higher explicit emotional cognition). This model also includes other regions such as amygdalae which seems to be associated with the affective and cognitive dimensions of alexithymia via its respective links with the prefrontal cortex and neocortical regions.

The identified regions are involved in emotional processes. To start with, regarding white matter involvement, MS constitutes a model of “multiple disconnection syndrome” arising from demyelination and axonal loss in several white matter tracts depending on the lesions’ location and extent (Chalah and Ayache, 2024). The CC constitutes the largest interhemispheric white matter bundle, the atrophy of which would result in a deficiency in interhemispheric transfer. The atrophy of other cerebral and cerebellar white matter would disturb several tracts that take part in cognitive and affective networks. In addition, the involvement of cerebellar tracts provides additional evidence on the implication of the cerebellum, not only in motor processes but also in cognitions and emotions, including alexithymia (Laricchiuta et al., 2015). With regards to deep gray matter, the thalamus is a component of the limbic system, a relay center involved in the processing and integration of sensory, motor, cognitive and affective information. Its implication in alexithymia seems to occur in MS as well as in other populations (Goerlich-Dobre et al., 2015). The amygdala plays a key role in emotion perception, fear conditioning, reward learning, and social behavior (Xu et al., 2018). The relationship between alexithymia and left orbital IFG would suggest a less efficient reward, emotional evaluation and regulation (Xu et al., 2018). Via their connections with orbitofrontal, prefrontal, and cingulate regions, the basal ganglia are involved in emotions, including the crude distinction in global valence states and automatic behavioral display of expressions (Messina et al., 2014; Chalah et al., 2020a). Finally, brainstem involvement seems to be linked with emotional dysregulation (Capet et al., 2021).

## 4 Current knowledge and future perspectives

The available literature is scarce and faced with several limitations including the small sample size, the cross-sectional design, the lack of healthy control groups, the lack of subgroup analysis or covariates control. Yet, they confirm previous results on the high prevalence of alexithymia in MS and provide preliminary evidence on the structure and function of the alexithymic brain in the context of this disease. This seems to involve cortical gray matter (orbital IFG), deep gray matter (amygdala, thalami, basal ganglia), and several white matter tracts (CC, cerebellar and cerebral white matter). Further characterization of alexithymia in MS is needed to better understand its mechanisms and impact and be able to develop adequate and targeted interventions.

In terms of pathophysiology, it remains formally unclear whether alexithymia in MS is only related to neuropathological processes and/or chronic and unpredictable stress associated with the disease. The absence of differences in the prevalence of alexithymia among disease subtypes or stages goes against the latter point (Capet et al., 2021). In addition, alexithymia total scores appear to be globally stable over a 5-year follow-up period (Chahraoui et al., 2014). Future longitudinal multi-modal research with neuropsychological, neurophysiological (e.g., TMS-derived cortical excitability measures, high-resolution electroencephalography, specific autonomic nervous system assessment) and neuroimaging data (e.g., resting state and task-related fMRI, volumetry, diffusion tensor imaging, spectroscopy) could help answer this question. Here, research could also benefit from focusing on specific regional abnormalities within neural hubs with potential involvement in alexithymia (e.g., CC subparts as in Pelletier et al., 1996; Capet et al., 2021). Other regions of interest (insula, cingulate cortex) that have been linked to alexithymia in general but not in MS studies deserve to be further explored. In addition, while some authors considered the cognitive and affective dimensions of alexithymia and suggest the presence of different alexithymia subtypes (Larsen et al., 2003; Bermond et al., 2006, 2007), others suggest alexithymia to be a single-dimensional trait but with a variable extent of severity (Bagby et al., 2021). Moreover, the affective and cognitive alexithymia dimensions could be associated with distinct neural substrates (Goerlich-Dobre et al., 2014a, 2015; van der Velde et al., 2014). Therefore, it might be interesting to tackle this issue in future MS works. Furthermore, interoceptive deficits have been suggested to be associated with alexithymia in general (Brewer et al., 2016; Van Bael et al., 2024), and have been linked with some MS manifestations (Chalah and Ayache, 2024). Therefore, its involvement in alexithymia and other MS symptoms (anxiety, depression, fatigue), merit to be explored.

Therapeutically, there is no current consensus on management options. The potential utility of psychotherapy has been suggested ever since the early work of Freyberger (1977). Therapies specifically targeting alexithymia appear to be better suited (Cameron et al., 2014). Emotionally focused interventions could act on alexithymia, illness perception, and/or quality of life (Luca et al., 2024). Dialectical behavioral therapy-based interventions could help alleviate alexithymia and improve emotional identification (Salles et al., 2023). Besides psychotherapy, cognitive rehabilitation training involving the theory of mind has been found to reduce alexithymia in a small study done in MS, especially on the DDF component (d'Arma et al., 2023). In addition, brain stimulation techniques might have their utility. The current data is limited to one case report applying transcranial direct current stimulation in a patient with MS and alexithymia (Chalah et al., 2017a,b). Also, neurofeedback has been applied in alexithymia in general (Samur et al., 2013) as well as in PwMS regardless of alexithymia (Ayache et al., 2021), and its utility in PwMS exhibiting this construct merits to be tested. Antidepressants, particularly specific serotonin and noradrenaline reuptake inhibitors were found to decrease alexithymia in a one randomized trial on post-stroke depression and might present benefits if tested in MS (Cravello et al., 2009). Other experimental tools could include interoceptive technologies and treatment trials (e.g., intranasal oxytocin), but no studies have yet been conducted in MS (Samur et al., 2013; Schoeller et al., 2024). Admitting the frequency and potential impact of alexithymia in PwMS, these modalities applied alone or in combination merit to be explored aiming to pave the way for new therapeutic venues and improve patients’ quality of life.
